# Experimental Investigation of Mechanical Behaviors of Self-Compacting Concrete under Cyclic Direct Tension

**DOI:** 10.3390/ma12071047

**Published:** 2019-03-29

**Authors:** Zhi Shan, Zhiwu Yu, Feng Chen, Xiao Li, Jing Gao

**Affiliations:** 1 School of Civil Engineering & National Engineering Laboratory for High Speed Railway Construction & Engineering Technology Research Center for Prefabricated Construction Industrialization of Hunan Province, Central South University, 68 South Shaoshan Road, Changsha 410075, China; zhishan@csu.edu.cn (Z.S.); zhwyu@csu.edu.cn (Z.Y.); iamlixiao@csu.edu.cn (X.L.); 2 Sanneng Housing, Shaping Business Center, No. 6, Section 1, Middle Furong Road, Changsha 410008, China; 18774841994@163.com

**Keywords:** self-compacting concrete, cyclic direct tension, stress-stain curve, energy dissipation, damage model

## Abstract

Self-compacting concrete (SCC) is increasingly applied in the construction industries due to its advantages of outstanding workability and eco-friendliness. However, few experimental studies on the mechanical behaviors of SCC under cyclic direct tension are available in the literature. In this work, experimental investigations of mechanical behaviors of SCC under cyclic direct tension were conducted. Especially, direct tensile load equipment was developed. It is found that the envelope stress-strain curve under cyclic direct tension is close to that under monotonic direct tension; however, it is different from that in a compressive case. It is also revealed that the ratio of unloading strain to irreversible strain is approximately linearly dependent on unloading strain. The evolutions of energy dissipation for SCC under both cyclic direct tension and compression are similar to those of normally vibrated concrete. In addition, Poisson’s ratio is observed to be nearly 0.21 for SCC. Furthermore, a damage model enabling characterization of the stress-strain curve under both monotonic and cyclic loading was proposed and verified against experimental results. Therefore, the results in this work provide original research material for studying and modelling the mechanical behaviors of SCC under uniaxial cyclic direct tension.

## 1. Introduction

Concrete materials have played an essential role in the development of built environments and civilizations for more than one hundred years. Recently, a new type of high performance concrete named self-compacting concrete (SCC) was developed and is being increasingly used in the construction industries due to its inherent advantages of outstanding workability, i.e., high fluidity, good segregation resistance, and distinctive self-compacting ability. Specifically, during the casting process, SCC is capable of flowing through and filling gaps of reinforcement and corners of molds, compacting with excellent homogeneity and presenting no segregation, by requiring no external vibration [1,2]. Subsequently, this outstanding workability helps SCC to possess higher strength, better durability, and more eco-friendly qualities compared to normally vibrated concrete (NVC) [2].

In order to have a better understanding of the mechanical behaviors for further conducting relevant applications on buildings and structures with SCC, a number of celebrated works have been contributed by researchers, mainly concerning the mechanical properties of SCC under monotonic loading. For example, Brouwers and Radix [3] conducted a series of experimental and statistical studies on the compressive strength and splitting tensile strength of SCC under uniaxial loading. Filho, et al. [4] observed the compressive strength, splitting tensile strength, and Young’s modulus of SCC under uniaxial loading. Furthermore, several works were also only limited to investigations on the properties of SCC under monotonic loading, e.g., the compressive/tensile strength [5,6,7,8], Young’s modulus [5,6], constitutive relationship under uniaxial compression [7], and fracture property [8].

Nevertheless, a few experimental results of the mechanical behaviors of SCC under cyclic direct tension have been reported in the literature [2,3,4,5,6,7,8,9,10,11,12,13]. Specifically, initially, several researches focused on studying mechanical behaviors under cyclic compression. For example, Fathi and Farhang [9] experimentally observed the effect of cyclic compressive loading on the heated SCC, and reported the results of stress-strain curves. Fathi and Dabbagh [10] experimentally studied the effect of cyclic compressive loading at different frequencies on SCC, with a focus on the unloading and reloading curves. In addition, although the mechanical behaviors of SCC under fatigue loading have been observed by several researchers [2], the observations were restricted to considering the effects of train loading on the mechanical behaviors. The fatigue loading due to a train typically leads to a lower strain amplitude and higher cyclic number limit of the materials than that resulting from cyclic loading, for example, earthquakes. Furthermore, when related to the mechanical behaviors under tension, only experimental results of splitting tensile strength tests have been reported in the literature [3,4,5]. Therefore, other mechanical behaviors, e.g., stress-strain curves, under cyclic tension are still unavailable.

However, due to the purpose of conducting a safe and economic analysis and designing of buildings and structures, knowledge of mechanical behaviors of SCC under cyclic direct tension is in urgent need, when considering the vastly growing usage of SCC in earthquake regions across the world. The detailed reasons for this are as follows. Firstly, the mechanical behaviors under cyclic direct tension are a fundamental aspect of the material properties. Secondly, the rebar reinforcements generally result in a sophisticated stress status, including tension in typical SCC structures. Besides, the earthquake loads usually cause the appearance of cyclic tension in the structures.

Therefore, in this work, the mechanical behaviors of SCC under cyclic direct tension are investigated experimentally. The outline of this work is as follows. After the materials and methods are obtained by initially introducing direct tensile load equipment, the experimental investigations of the mechanical behaviors under cyclic direct tension are conducted, including the stress-strain curve and evolution of energy dissipation. Then, a damage model for expressing the stress-strain curves under both the monotonic and cyclic loading is proposed.

## 2. Materials and Methods

### 2.1. Materials

In order to investigate the mechanical behaviors of the SCC under cyclic direct tension, a total of 46 specimens were considered with a mixture ratio according to the technical specification in China [14]. The mixture ratio was designed as cement:fly ash:mineral powder:expanding agent:sand:aggregate:water:admixture = 331:108:64:48:824:823:177:5.5 (unit: kg/m^3^). In detail, the cement is Portland cement of P.II 42.5 R, the water is tap water, the aggregates are crushed limestone with diameters of approximately 5–20 mm, the sands are middle grain sands, the mineral admixtures are the mineral powder of S95 and fly ash, and the admixture is the water-reducing agent of NoF-II.

The workability of SCC was tested after the technical specification [14] and is listed in Table 1. The results show that the material meets the relative requirements.

### 2.2. Specimens

The dimensions of the prismatic specimens were designed as 100 × 100 × 300 mm^3^, and the ones of the cube specimens were designed as 150 × 150 × 150 mm^3^. All the specimens were cured by the standard method for 28 days [14]. The specimens used for the tests were listed as follows: 14 cube specimens were tested for determining the compressive strength, nine cube specimens were tested for obtaining the stress-strain curves under monotonic compressive loading, 13 prismatic specimens were tested for determining the stress-strain curves under monotonic direct tensile or compressive loading, and 10 prismatic specimens were tested for determining the stress-strain curves under cyclic direct tensile or compressive loading.

### 2.3. Experimental Setup

**Figure 1:**
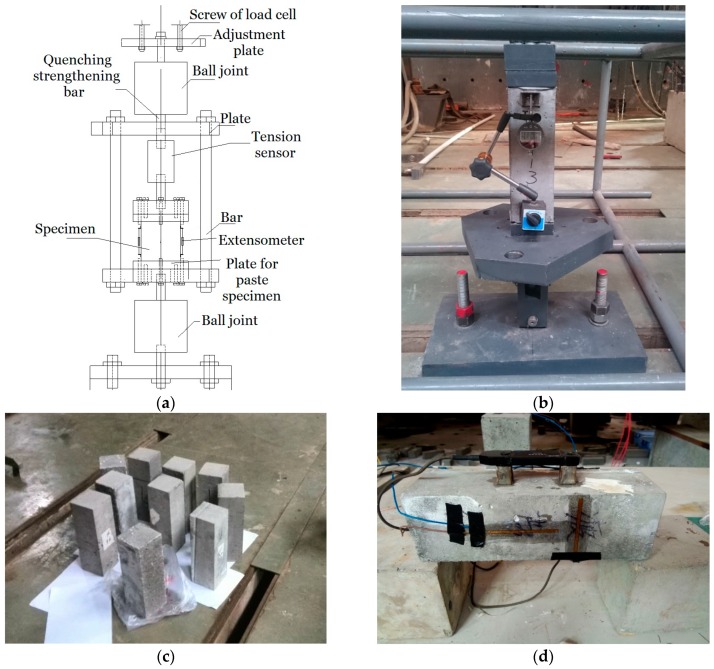
Experimental setup and SCC specimens. (**a**,**b**) Direct tensile load equipment. (**c**) A picture of specimens after curing. (**d**) The layout of the electrical resistance strain gauges and the extensometer.

Both the uniaxial monotonic and cyclic direct tension tests were conducted in a MTS-810 hydraulic servo testing machine (maximum capacity of 250 kN with a resolution of ±0.5%, MTS Systems Corporation, Eden Prairie, MN, USA) by applying direct tensile load equipment, as shown in Figure 1a,b. The equipment consists of several components, as follows (Figure 1a): an adjustment plate for adjusting the equipment position, two ball joints that enable equal-load sharing and balance the cracking specimen, two plates for pasting specimen, bars for sharing the unloaded forces from the specimen, a tension sensor, and an extensometer, etc. In detail, the adjustment plate was used for adjusting the equipment position by mainly considering the position of the load cell and the size of the specimen. The ball joints were fixed close to the top and bottom of the equipment (the adjustment plate and the foundation, respectively). They are essential for ensuring the success of tensile tests, since they are able to help the tensile load to equally subject on the intact part of a specimen after cracks are initiating. The plates for pasting the specimen were designed to directly subject tension to the specimen, and the adhesive for structural bonding used to paste the plates and the specimen is type JGN-II with a minimum tensile strength of 35 MPa, and the setting time is approximately 24–48 h. No bonding failures were observed during all the experiments, which verified the efficiency of the adhesives we selected. The bonding area was located out of the measurement range of the extensometer, for the purpose of avoiding the effects of the deformation resulting from this area. The bars were able to maintain a slow loading rate to ensure that the post-peak deformation/force was measurable for the specimen, by sharing the unloaded forces from the specimen after cracks developed. The tension sensor was applied for detecting the value of the tensile load subjected on the specimen with a resolution of 0.005 kN. The extensometer was applied for testing the specimen deformation with a resolution of 0.001 mm.

The procedure of the monotonic tension test was as follows [15]. A test was first conducted for determining the direct tensile strength *f*_t0_. During the experiments for testing the stress-strain curves, the loading pattern was selected as the force-controlled method by increasing with a constant rate of 2 kN/min in the range of 0–0.9*f*_t0_, and then changed to the displacement-controlled method with a rate of 0.0005 mm/s, until the monitored force was decreased to approximately 0.1*f*_t0_.

The procedure of the cyclic tension test was as follows [15]. In the range of 0–0.8*f*_t0_, the loading pattern was selected as the force-controlled method by increasing with a constant rate of 2 kN/min until each unloading point, which was determined as 0.2*f*_t0_, 0.4*f*_t0_, 0.6*f*_t0_, and 0.8*f*_t0_. Then, unloading to 0.01*f*_t0_ was conducted to avoid the compression due to the gravity load of the equipment on the specimen. After the loading stress was larger than 0.8*f*_t0_, the loading pattern was changed to the displacement-controlled method with a rate of 0.0005 mm/s, and the determination of the unloading points in this range was determined by a trial and error method, until the monitored reloading force was decreased to approximately 0.1*f*_t0_.

Both the uniaxial monotonic and cyclic compression tests were carried out in a servo-hydraulic universal testing machine (INSTRON 1346, maximum capacity of 2000 kN with a resolution of ±0.5%, Instron Corp, Buckinghamshire, HP, UK), according to the code [15]. The strains/ deformations of each specimen were monitored by adopting both the electrical resistance strain gauges with a resolution of 1 *με* and the extensometer (Figure 1).

## 3. Results and Discussion

### 3.1. Direct Tensile Strength

The experimental results of direct tensile strength of SCC prismatic specimens are listed in Table 2. By using the statistical analysis method [15], the mean tensile strength of prismatic specimens was obtained as *f*_tm_ = 3.29 MPa. Additionally, the experimental results of the compressive strength of cube specimens are listed in Table 3. The mean compressive strength of cube specimens was computed as *f*_cm_ = 39.94 MPa, and the coefficient of variation (CV) of compressive strength was obtained as *δ* = 0.129. It was found that the calculated ratio of the mean tensile strength to the mean compressive strength (*f*_tm_/*f*_cm_) was 0.093, which is higher than that of NVC, which was normally around 0.075.

### 3.2. Stress-Strain Curve under Monotonic Direct Tension

**Figure 2:**
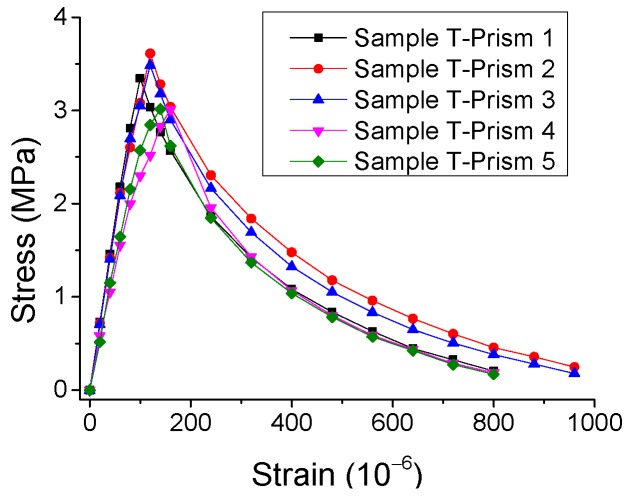
Experimental results of stress-strain curves for SCC prismatic specimens under monotonic direct tension.

**Figure 3:**
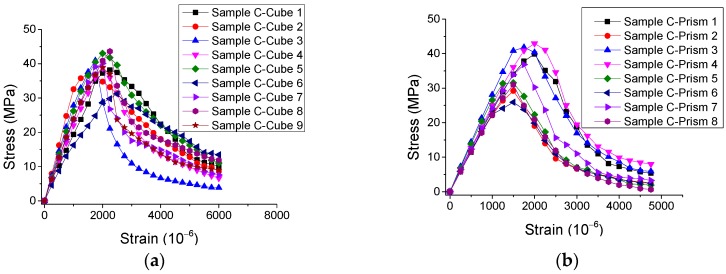
Experimental results of stress-strain curves for SCC under monotonic compression. (**a**) Cube specimens and (**b**) prismatic specimens.

**Figure 4:**
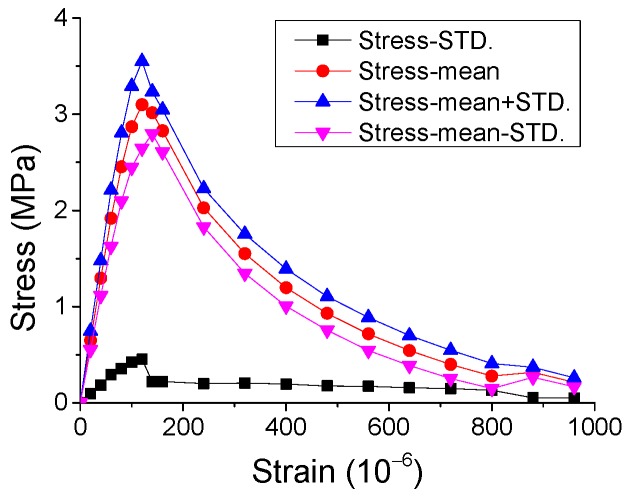
Mean and STD. of stress-strain response of SCC prismatic specimens under monotonic direct tension.

**Figure 5:**
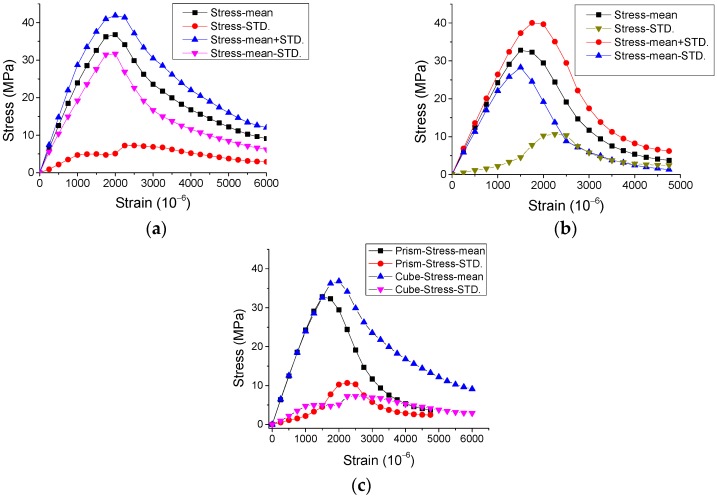
Mean and STD. of stress-strain response of SCC specimens under monotonic compression. (**a**) Cube specimens, (**b**) prismatic specimens and (**c**) results comparison of cube and prismatic specimens.

The experimental results of the stress-strain curves under monotonic direct tension and compression are represented in Figure 2 and Figure 3, with the statistical analysis of the results plotted in Figure 4 and Figure 5.

**Figure 6:**
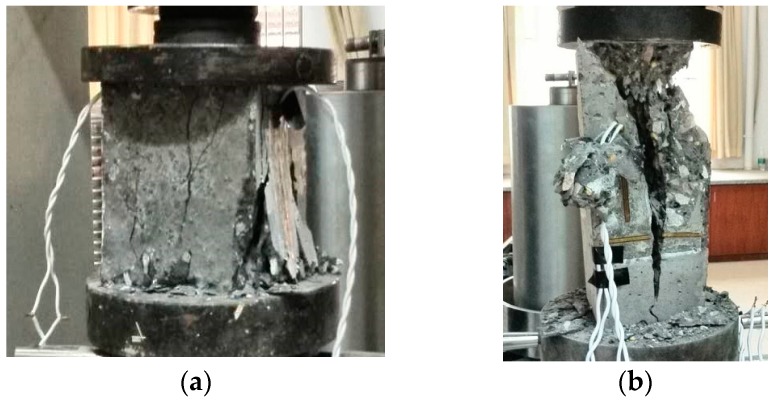
Failure modes of SCC under monotonic compression. (**a**) Cube specimen and (**b**) prismatic specimen.

By observing the results in Figure 2, Figure 3, Figure 4 and Figure 5, it is revealed that the SCC shows a stochastic stress-strain response under both uniaxial monotonic direct tension and compression. In Figure 4, the strain corresponding to the peak point in the standard deviation (STD.) curve is close to that in the mean stress-strain curve under monotonic direct tension. By contrast, for the monotonic compression case in Figure 5, the strain corresponding to the peak point in the STD. curve is significantly higher than that in the mean stress-strain curve. Additionally, comparing Figure 4 with Figure 5, it is found that the mean stress-strain curve under monotonic direct tension shows a significantly more brittle response. Furthermore, under monotonic compression, the mean peak stress of the prismatic specimens is evidently lower than that of the cube specimens, since the confinement effect due to friction between the load cell and surface of the prismatic specimen is generally lower than that of the cube one (Figure 6).

### 3.3. Stress-Strain Curve under Cyclic Direct Tension

**Figure 7:**
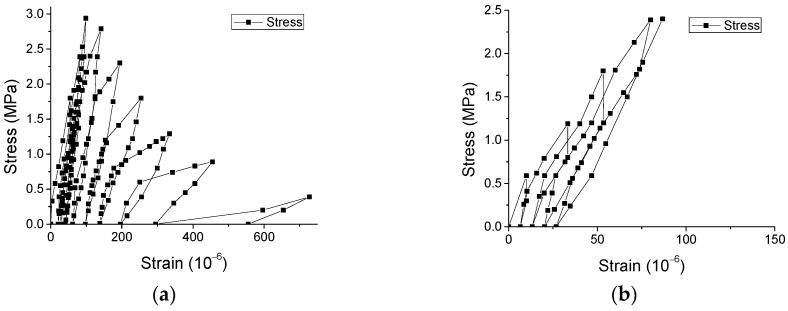
Typical experimental results of stress-strain curves for SCC prismatic specimens under cyclic direct tension. (**a**) Sample T-Prism 6 and (**b**) Sample T-Prism 7.

**Figure 8:**
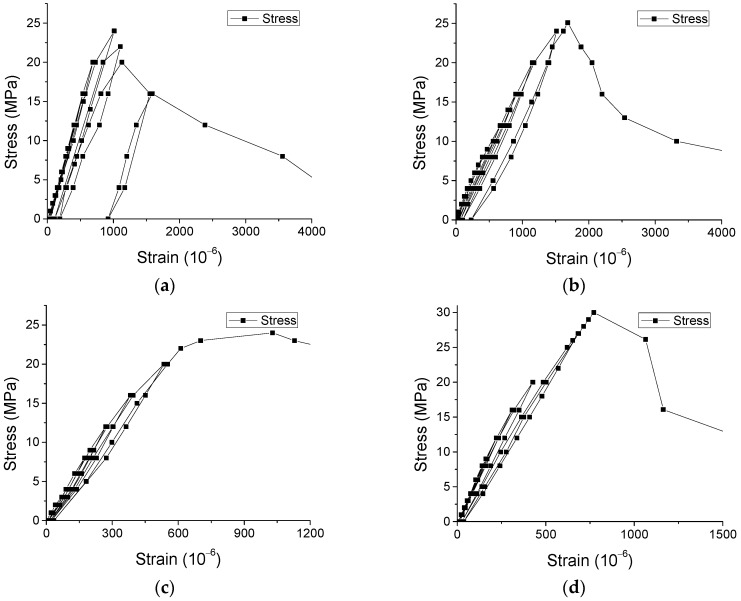
Typical experimental results of stress-strain curves for SCC prismatic specimens under cyclic compression. (**a**) Sample C-Prism 9, (**b**) Sample C-Prism 10, (**c**) Sample C-Prism 11, and (**d**) Sample C-Prism 12.

**Figure 9:**
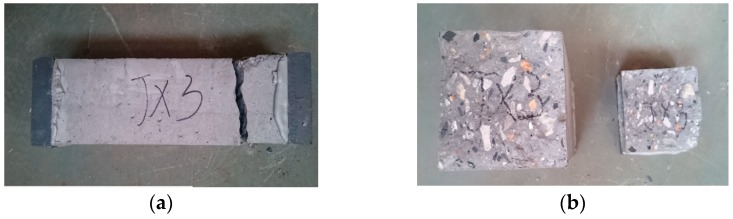
Failure modes of SCC prismatic specimens under cyclic direct tension. (**a**) Crack positions and (**b**) crack surfaces.

The experimental results of the stress-strain curves of SCC prismatic specimens under both cyclic direct tension and compression were obtained and are presented in Figure 7 and Figure 8. Additionally, the typical failure modes of the specimens under cyclic direct tension are illustrated in Figure 9. The observations are summarized as follows.

**Figure 10:**
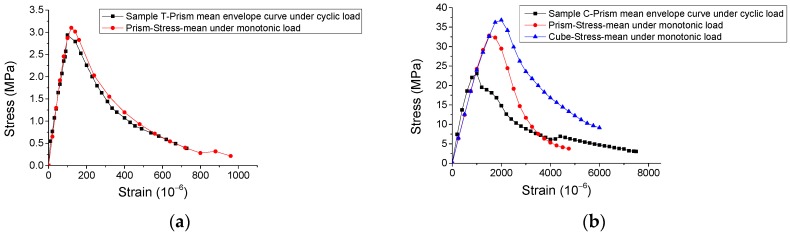
Comparison between envelope and monotonic stress-strain curves for SCC. (**a**) Direct tensile case and (**b**) compressive case.

Initially, Figure 10a illustrates that the envelope curve of the mean stress-strain response for SCC under cyclic direct tension is close to that under monotonic direct tension. By contrast, in Figure 10b, the envelope curve of the mean stress-strain response for the prismatic specimens under cyclic compression does not pass through that for the monotonic compression case, and the mean peak stress is nearly 15% lower than that in the monotonic case.

It is noted that there is no agreement about the relationship between the envelope of cyclic stress-strain curve and monotonic stress-strain curve. For example, Sinha et al. [16] reported that for the experimental results of NVC under cyclic loading, the envelope curve does not pass through the stress-strain curve in the monotonic loading case. However, Fathi and Farhang [9] observed different results, showing that both curves are close to each other for SCC in the experiment. This may be attributed to the different micro structures of the specimens caused by the different mixture ratio, raw materials, etc., for different types of concretes.

**Figure 11:**
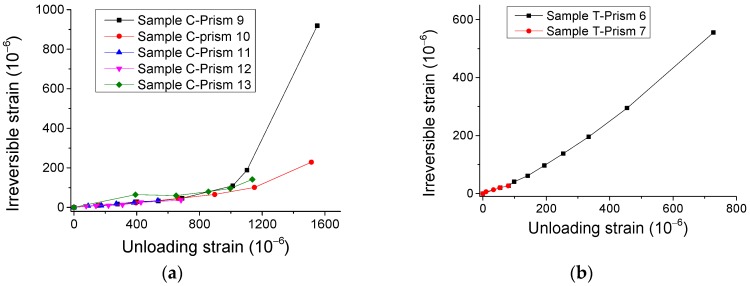
Experimental results of relationship of irreversible strain—unloading strain. (**a**) Compressive case and (**b**) direct tensile case.

**Figure 12:**
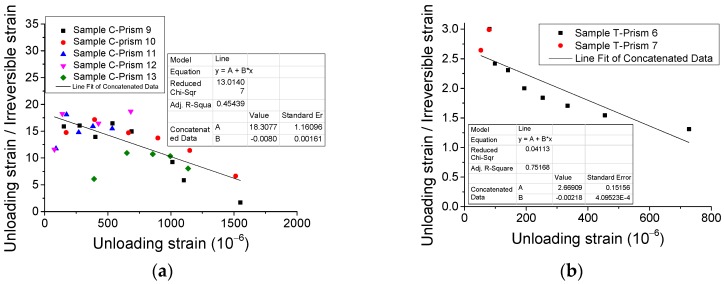
Relationship of unloading strain/irreversible strain–unloading strain. (**a**) Compressive case and (**b**) direct tensile case.

Additionally, Figure 11 shows that the irreversible strains nonlinearly rise with the growing of strains in the unloading points (unloading strains). However, based on further analysis of relevant variables, Figure 12 illustrates that the ratio of unloading strain to irreversible strain is approximately linearly dependent on unloading strain. It may be used for analyzing the irreversible/plastic strain for concrete, which is generally a concern of researchers [17].

### 3.4. Energy Dissipation under Cyclic Direct Tension

**Figure 13:**
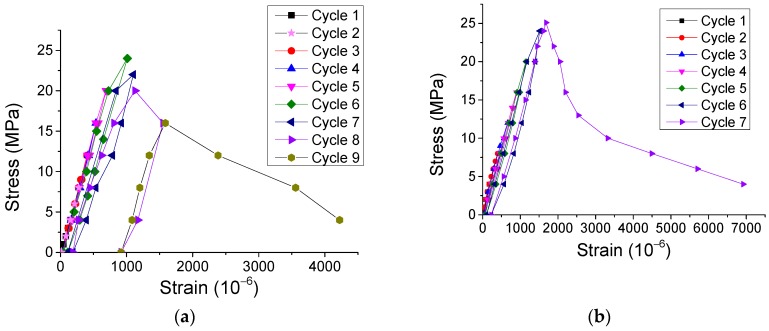

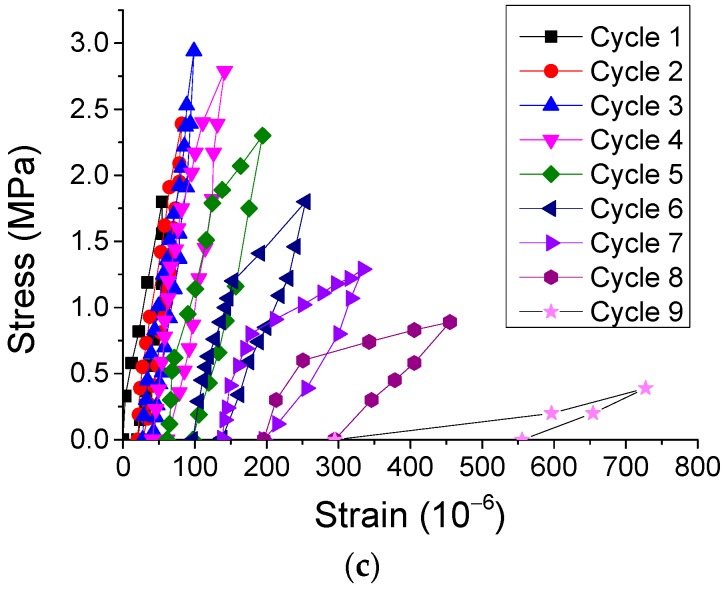
Typical experimental results of stress-strain curves of SCC considering the cycle number. (**a**) Sample C-Prism 9 in compressive case, (**b**) Sample C-Prism 10 in compressive case, and (**c**) Sample T-Prism 6 in direct tensile case.

**Figure 14:**
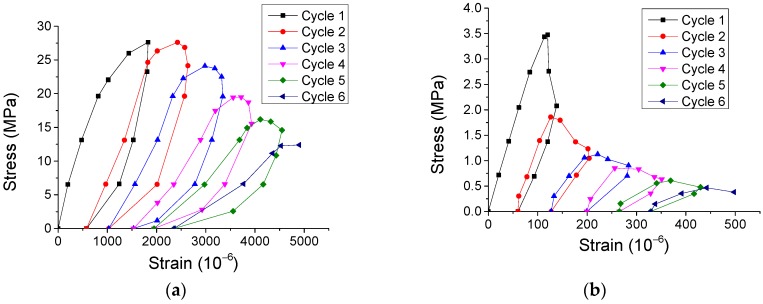
Typical experimental results of stress-strain curves of NVC considering the cycle number from literature. (**a**) Compressive case [19] and (**b**) tensile case [20].

**Figure 15:**
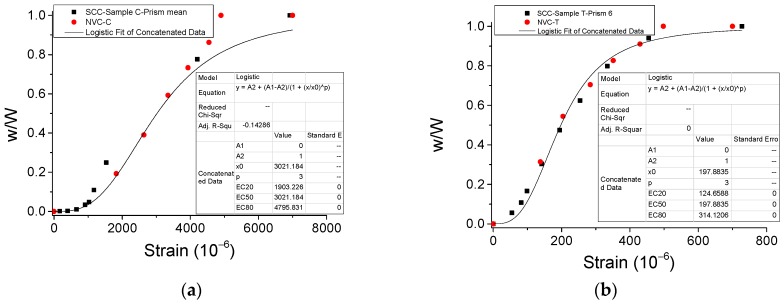
Evolution of normalized energy dissipation: *w*/*W* v.s. *ε*. (**a**) Compressive case and (**b**) tensile case.

The energy dissipation of materials under external loading is an essential aspect related to the mechanical properties [18]. In this work, in order to analyze the energy dissipation of SCC under cyclic direct tension/compression and conduct comparison with NVC, both typical experimental results of stress-strain curves considering the cyclic number from our study and literature [19,20] were plotted in Figure 13 and Figure 14. Further, a new variable, named normalized energy dissipation (*w*/*W*), was defined as the ratio of the sum energy area of current cycles to that of total cycles, where *w* = *A*_Cycle 1_ + *A*_Cycle 2_ +*A*_Cycle 3_ + … + *A*_Cycle *n*_, *W* = *A*_Cycle 1_ + *A*_Cycle 2_ +*A*_Cycle 3_ + … + *A*_Cycle *N*_. Here, *A* denotes the area inside the stress-strain curve and the *x*-axis for each cycle (Figure 13 and Figure 14), and *n* and *N* denote the number of current cycle and total cycles, respectively. Therefore, the evolution of the normalized energy dissipation was obtained in Figure 15. It illustrates that the evolution of both SCC and NVC under cyclic loading experiences a three-stage process. Precisely, after the normalized energy dissipation increases slowly with the growth of strain at the beginning, it rapidly grows during the second stage, and finally smoothly approaches 1.0 during the last stage. Additionally, Figure 15 also shows that the normalized energy dissipation evolution of SCC is close to that of NVC under both cyclic compression and tension, respectively. The fitting models of the evolution were also obtained in Figure 15.

### 3.5. Poisson’s Ratio under Monotonic Compression

**Figure 16:**
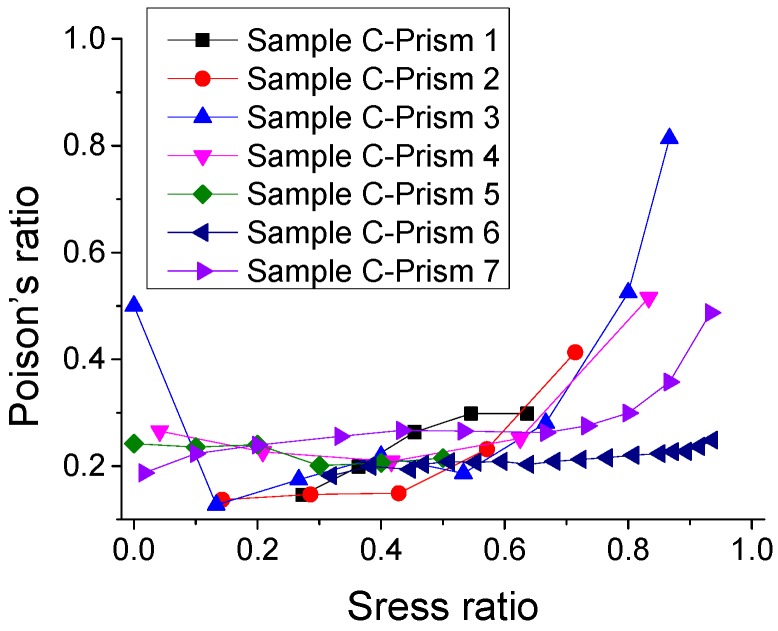
Experimental results of relationship of Poisson’s ratio–stress ratio (*υ* v.s. *σ*/*f*_c_).

**Figure 17:**
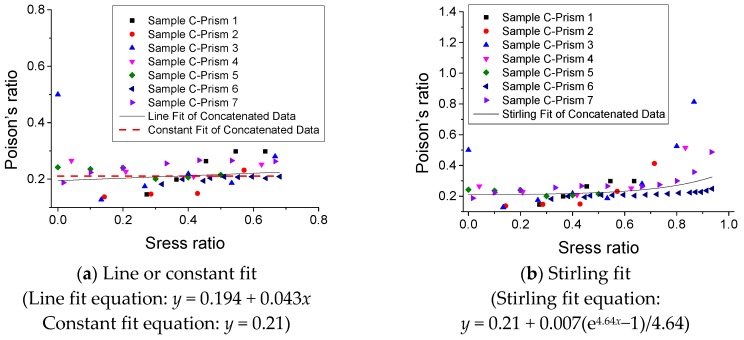
Fitting model and its predicted results of relationship of Poisson’s ratio - stress ratio (*υ* v.s. *σ*/*f*_c_). (**a**) Line or constant fit and (**b**) Stirling fit.

The experimental results of Poisson’s ratio (*v*) of SCC under monotonic compression are described in Figure 16. It is observed that Poisson’s ratio remains approximately constant as 0.21 in the range of the stress ratio (*σ*/*f*_c_) 0–0.7, and then increases as the stress ratio increases. Moreover, Figure 17 shows three fitting models and the relative predicted results of the relationship of Poisson’s ratio–stress ratio. It is suggested that for SCC, the value of Poisson’s ratio *υ* = 0.21 is able to be used for approximate calculation in certain cases. However, when it requires an accurate analysis for SCC structures, a more precise model (e.g., a model in Figure 17b) is needed.

### 3.6. Damage Model for Stress-Strain Curve

Among a number of damage models [17,21,22,23], a fiber bundle-plastic chain model (BCM) [21] is developed based on the classical fiber bundle model (FBM) for characterizing the mechanical behaviors of quasi-brittle materials under external loading. The damage model BCM was verified to be able to capture the stochastic micro structure and the crack/damage behaviors of quasi-brittle materials and describe the stress-strain curve under cyclic loading precisely and conveniently [21,22]. Therefore, the suggested damage model equation is based on BCM [21,22], as given in the following equations:

(1) $$\sigma =\left[1-dr\left(\varepsilon \right)\right]\cdot E_{0}\cdot \varepsilon ,$$

(2) $$\varepsilon_{i}=r\left(\varepsilon \right)\cdot \varepsilon ,$$

(3) $$E=\frac{1-dr\left(\varepsilon \right)}{1-r\left(\varepsilon \right)}\cdot E_{0},$$

(4) $$dr\left(\varepsilon \right)=\frac{A_{1}-A_{2}}{1+{\left(\varepsilon /\varepsilon_{0}\right)}^{p}}+A_{2},$$

(5) $$\frac{1}{r\left(\varepsilon \right)}=B_{1}+B_{2}\cdot dr\left(\varepsilon \right)+B_{3}\cdot {\left[dr\left(\varepsilon \right)\right]}^{2}.$$

where *E*_0_ and *E* denote the initial and current Young’s modulus respectively; *ε*_i_ denotes the irreversible/plastic strain; and *dr*(*ε*) and *r*(*ε*) denote the plastic-damage variable and plastic variable, respectively, considering the effect of the material micro-structure and external loads [21]. Additionally, *A*_1_, *A*_2_, *ε*_0_, and *p* denote the parameters relating to the damage/plasticity of the material due to the stochastic properties of its micro-structure [21]; *B*_1_, *B*_2_, and *B*_3_ are the parameters relative to the coupling of damage and plasticity [22]; and the parameters are able to be calibrated by fitting experimental results.

**Figure 18:**
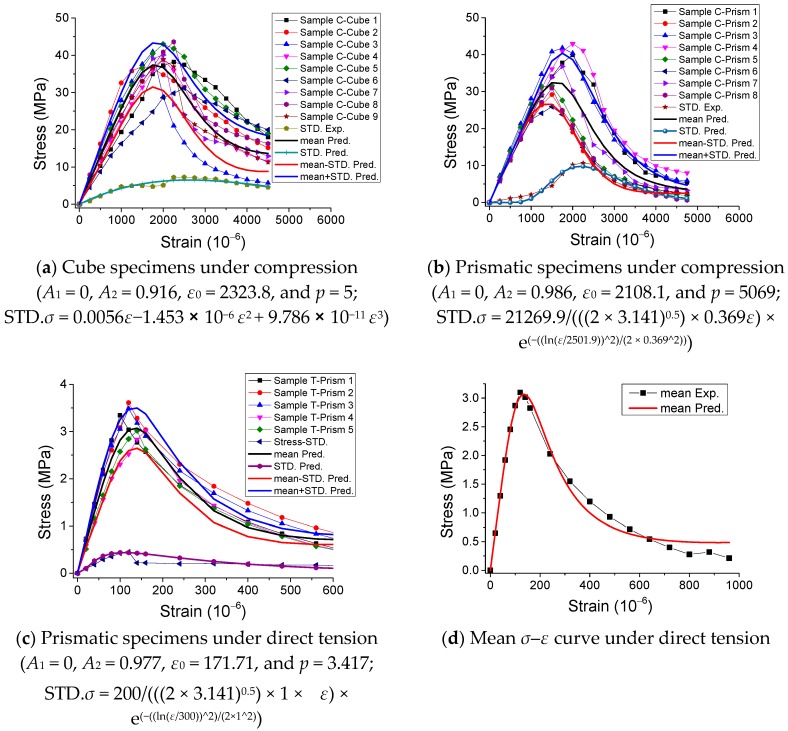
Stress-strain curves of SCC under monotonic loading. (**a**) Cube specimens under compression, (**b**) Prismatic specimens under compression, (**c**) Prismatic specimens under direct tension and (**d**) Mean *σ-ε* curve under direct tension.

**Figure 19:**
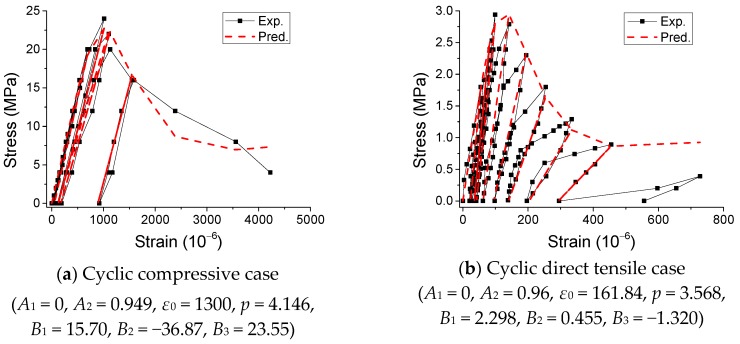
Stress-strain curves of SCC under cyclic loading. (**a**) Cyclic compressive case and (**b**) Cyclic direct tensile case.

Furthermore, the predicted results based on the suggested equation were obtained in Figure 18 and Figure 19 concerning the stress-strain curves. Figure 18 shows that the predicted results agree with the experimental results of stress-strain curves under monotonic loading. In addition, Figure 19 illustrates that the predicted results coincide with the experimental results of stress-strain curves under cyclic loading. Hence, the suggested equation is able to characterize the mechanical behaviors of SCC under both monotonic and cyclic loading.

## 4. Conclusions

In this work, experimental investigations of the mechanical behaviors of SCC under uniaxial cyclic direct tension and compression were reported. The direct tension tests were conducted by using direct tensile load equipment developed by the authors’ research team. The following conclusions were able to be obtained.

The envelope of the stress-strain curve for SCC under cyclic direct tension is close to the stress-strain curve under monotonic direct tension; however, the envelope under cyclic compression does not pass through that under monotonic compression. In the current academic community, there is no agreement about the relationship between the envelope of the cyclic stress-strain curve and monotonic stress-strain curve. It may be attributed to the different micro structures of the materials caused by different mixture ratios, raw materials, etc., for different types of concretes. Under cyclic loading, the different micro structures of the materials may generate different patterns of crack initiation, growth, and frictions between the cracks, further resulting in various deteriorating processes, including strength degradation, strain-stress responses, etc.

Additionally, the ratio of unloading strain to irreversible strain is approximately linearly dependent on unloading strain. Poisson’s ratio was found to be nearly 0.21, which is suggested to be used for approximant calculation. The evolution of energy dissipation of SCC under cyclic direct tension and compression is close to that of NVC. The reason for this is that the energy dissipation for materials is generally contributed by the total changes of the micro structures due to external loading, including the crack initiation, growth, and frictions between the cracks. By considering that both the SCC and NVC are quasi-brittle materials, the evolutions of the energy dissipation caused by the above-mentioned factors for these two materials are similar.

Furthermore, a damage model for characterizing the stress-strain curve of SCC under both monotonic and cyclic loading was also proposed. It was verified by comparing the predicted results with the experimental results.

This work may be of significance for studying and modelling the mechanical behaviors of SCC under uniaxial cyclic direct tension, and providing a better analysis and design of SCC structures.

## Figures and Tables

**Table 1:** Workability test results of SCC.

Test	Slump Flow *S*_f_/mm	Slump Time *T*_50_/s	Height of J-ring *H*_J_/mm	Height Ratio of L-box *H*_2_/*H*_1_
Result	650	4.5	15	0.9

**Table 2:** Tensile strength *f*_t_ of prismatic specimens (MPa).

No.	T-Prism1	T-Prism2	T-Prism3	T-Prism4	T-Prism5
Strength *f*_t_	3.345	3.612	3.484	3.011	3.017

**Table 3:** Compressive strength *f*_c_ of cube specimens (MPa).

No.	C1	C2	C3	C4	C5	C6	C7	C8	C9	C10	C11	C12	C13
Strength *f*_c_	41.81	39.6	35.46	38.4	38.93	41.52	37.54	41.47	33.29	43.46	31.24	45.92	50.59
